# Episiotomy practices in France: epidemiology and risk factors in non-operative vaginal deliveries

**DOI:** 10.1038/s41598-020-70881-7

**Published:** 2020-11-19

**Authors:** Christophe Clesse, Jonathan Cottenet, Joelle Lighezzolo-Alnot, Karine Goueslard, Michele Scheffler, Paul Sagot, Catherine Quantin

**Affiliations:** 1 Centre for Psychiatry, Wolfson Institute of Preventive Medicine, Barts & The London School of Medicine & Dentistry, Queen Mary, University of London, Old Anatomy Building Charterhouse Square, London, EC1M 6BQ UK; 2 Interpsy Laboratory (EA 4432), Universite de Lorraine - Campus Lettres Et Sciences Humaines, Nancy, France; 3 Majorelle Polyclinic, Nancy, France; 4 Biostatistics and Bioinformatics (DIM), University Hospital, University of Burgundy and Franche-Comté, Dijon, France; 5 Psychologie Clinique, Interpsy Laboratory (EA4432), Lorraine University, Nancy, France; 6 Obstetricial Gynecologist, Endocrinologist, Gynecologist, The FNCGM (National Federation of Gynecology Medical Colleges), Cabinet de Gynécologie Médicale Et Obstétrique, 21 avenue Foch, 54000 Nancy, France; 7 Department of Obstetrics and Gynecology, University Hospital, Dijon, France; 8 Inserm, CIC 1432, Clinical Investigation Center, Clinical Epidemiology/Clinical Trials Unit, Dijon University Hospital, Dijon, France; 9 Biostatistics, Biomathematics, Pharmacoepidemiology and Infectious Diseases (B2PHI), INSERM, UVSQ, Institut Pasteur, Université Paris-Saclay, Paris, France

**Keywords:** Health care, Risk factors

## Abstract

Episiotomy use has decreased due to the lack of evidence on its protective effects from maternal obstetric anal sphincter injuries. Indications for episiotomy vary considerably and there are a great variety of factors associated with its use. The aim of this article is to describe the episiotomy rate in France between 2013 and 2017 and the factors associated with its use in non-operative vaginal deliveries. In this retrospective population-based cohort study, we included vaginal deliveries performed in French hospitals (N = 584) and for which parity was coded. The variable of interest was the rate of episiotomy, particularly for non-operative vaginal deliveries. Trends in the episiotomy rates were studied using the Cochran-Armitage test. Hierarchical logistic regression was used to identify variables associated with episiotomy according to maternal age and parity. Between 2013 and 2017, French episiotomy rates fell from 21.6 to 14.3% for all vaginal deliveries (p < 0.01), and from 15.5 to 9.3% (p < 0.01) for all non-operative vaginal deliveries. Among non-operative vaginal deliveries, epidural analgesia, non-reassuring fetal heart rate, meconium in the amniotic fluid, shoulder dystocia, and newborn weight (≥ 4,000 g) were risk factors for episiotomy, both for nulliparous and multiparous women. On the contrary, prematurity reduced the risk of its use. For nulliparous women, breech presentation was also a risk factor for episiotomy, and for multiparous women, scarred uterus and multiple pregnancies were risk factors. In France, despite a reduction in episiotomy use over the last few years, the factors associated with episiotomy have not changed and are similar to the literature. This suggests that the decrease in episiotomies in France is an overall tendency which is probably related to improved care strategies that have been relayed by hospital teams and perinatal networks.

## Introduction

Episiotomy is a “surgical enlargement of the vaginal orifice by an incision to the perineum during the last part of the second stage of labor or delivery”^1^. Nowadays, the use of this surgical procedure has decreased due to the lack of evidence associated with its protective effects against sphincter-related maternal complication induced by vaginal delivery^2–6^. Episiotomy rates vary considerably from one country to another (11.6% in the United States or 15.2% in England in 2012 compared with 75% in Cyprus, 67.5% in Poland in 2010, 68% in India in 2008 or 44.9% in Singapore in 2011)^3,7^. In 2005, which corresponds of the date of the publication of French guidelines for episiotomy^8^, the overall episiotomy rate in France was 41.3%^9^. The guidelines led to an immediate decline, resulting in an episiotomy rate of 35% in 2006^9^. The downward trend continued, and in 2010 the overall percentage of episiotomy was 27.1%^9,10^. In 2016, the most recent national perinatal survey reported a nulliparous rate of 34.9% (n = 4,083) and a multiparous rate of 9.8% (n = 5,899) with an overall rate of 20.1%)^11^.

Today, the medical indications for episiotomy vary considerably^12–14^. Recent studies have also shown the great variety of factors associated with episiotomy and their preponderance^15,16^. Furthermore, we still don’t know if the downward trend in episiotomy in some countries^3^ had an influence on the indication of episiotomy and its related risk factors. In this context, it seems relevant to provide regular updates concomitant with the overall statistical shifts in the practice of episiotomy. Recent studies have especially highlighted that maternal age^17–22^, parity^20,23–26^, and operative vaginal deliveries are associated with episiotomy^16^. In France, the latest national data^22,27^ and a more recent study^28^ have also associated episiotomy with nulliparity, advanced age of nulliparous mothers, high birth weight (> 4,000 g), breech vaginal deliveries, epidural analgesia, operative vaginal delivery, advanced gestational age, non-reassuring fetal heart rate (NRFHR) and multiple pregnancies. Then, in 2015–2016, a national debate on obstetric procedures including episiotomy emerged, making it essential to update the available data on episiotomy in France^29^. This debate is the consequence of a series of media events, which fueled a surge in social concerns around the conditions of delivery in France, and more generally around the question of the experience of health system users in gynecology and obstetrics. These events attracted the attention of the public authorities and prompted them to take an official position on the subject.

In this context, using French hospital data (including all private and public hospitals), we conducted a study to determine the epidemiological reality of episiotomy in France and the changes that occurred from 2013 to 2017 in the subgroup of non-operative vaginal deliveries, which are considered to be at a "reduced-risk" of episiotomy worldwide. The purpose of this article is first to describe the episiotomy rate in France between 2013 and 2017, and second, to study the factors associated with the use of episiotomy in the subgroup of non-operative vaginal deliveries.

## Methods

A retrospective population-based cohort study was carried out on all vaginal deliveries in French hospitals between 2013 and 2017. For this purpose, we used the French hospital database (PMSI), which collects anonymous individual data on medical and administrative information from all French private and public hospitals^30–33^ in a rigorous and standardized manner. The diagnoses are then coded according to the International Classification of Diseases (ICD-10), and the procedures are coded from the French Common Classification of Medical Procedures (CCMP). To ensure the quality of data collection, quality control procedures are carried out a posteriori on sample datasets by medical inspectors and territorial medical advisors.

### Inclusion and exclusion criteria

All vaginal deliveries for which parity was coded between 2013 and 2017 were identified by ICD-10 or CCMP codes (see Supplementary Table S1 online), from 584 hospitals, including 28 teaching hospitals.

Women under 12 or over 55 years old were excluded. We also excluded hospitals where no episiotomy was coded or that have no maternity unit and had reported only one delivery.

### Outcome and selected variables

The variable of interest was the rate of episiotomy (CCMP code JMP006) determined for vaginal deliveries for which forceps/spatula or vacuum extraction was not used (non-operative vaginal deliveries). Regarding the type of episiotomy, the French operators (like in most European countries) tend to perform mostly mediolateral episiotomies^1^.

In France, episiotomies associated with operative vaginal deliveries may not be systematically coded by the operator. Thanks to the data used in the validation study published by our team in the BMJ Open^34^, we found that the lack of coding of episiotomy in this group was less than 10% (8.7%). The under-estimation is therefore not very high, allowing us to estimate rates for all vaginal deliveries, but there is a risk of bias when conducting analyses on risk factors. For this reason we studied only risk factors for non-operative vaginal deliveries.

The variables included in our analyses were: hospital status (teaching/non-teaching), parity (nulliparous/multiparous), maternal age at delivery (< 20, 20–29, 30–39 and ≥ 40), year of discharge, prematurity (defined according to the WHO classification as a birth that occurred before 37 weeks of gestation^35,36^, diabetes mellitus (pre-existing or gestational diabetes), type of pregnancy (multiple/single), type of presentation (cephalic/breech), hypertensive disorders (including chronic hypertension, gestational hypertension, preeclampsia and eclampsia), epidural analgesia, non-reassuring fetal heart rate (NRFHR) related to labor and delivery complicated by an abnormal fetal heart rate (including fetal bradycardia, fetal heart rate irregularity and fetal tachycardia), meconium in the amniotic fluid, shoulder dystocia and scarred uterus(due to previous caesarean or previous surgery). To take into account the weight of newborns (< 4,000 g and ≥ 4,000 g), as a proxy of macrosomia, we also conducted an analysis on singleton deliveries. All ICD-10 or CCMP codes used to identify these variables are presented in Supplementary Table S1 online.

### Statistical analyses

In the descriptive analysis, the episiotomy rate was presented for all vaginal deliveries and then according to parity for all non-operative vaginal deliveries. We used the Cochran-Armitage test to evaluate trends in episiotomy rates between 2013 and 2017.

Concerning non-operative vaginal deliveries, we first compared rates of episiotomy for each modality of the variables studied. Qualitative variables were compared using the Chi-2 test. We then used two types of hierarchical regressions (with hospital-specific random intercept) according to the rarity of episiotomy to identify the factors associated with episiotomy and to take into account the variation in the episiotomy rate between centers. If the outcome was not rare (> 10%) then we used a hierarchical log binomial regression to calculate a relative risk, otherwise we used a hierarchical logistic regression to calculate an odds ratio^37^. The first level was related to individual maternal characteristics, nested with center (second level). For each hierarchical regression, we adjusted for patient characteristics. Logistic regressions were also performed for singleton deliveries according to the weight of newborns. Correlation and interaction were tested. As the different factors included in these regressions were different depending on parity and age, and because there were interactions between maternal age and parity, we decided to perform separate analyses depending on maternal age and parity.

All of the hypotheses were tested at an alpha risk of 0.05. Relative risks (RR) and odds ratios (OR) were given and 95% confidence intervals calculated. SAS version 9.3 (SAS Institute Inc., Cary, NC, USA) was used for statistical analyses. The methods were carried out in accordance with the relevant guidelines and regulations (Supplementary file online).

This study was approved by the National Committee for data protection (registration number 1576793). Written consent was not needed for this study as informed consent was waived by the ethics committee and as all data are anonymous. The data from the PMSI database was transmitted by the national agency for the management of hospitalization data (ATIH number 2015-111111-47-33).

## Results

**Figure 1:**
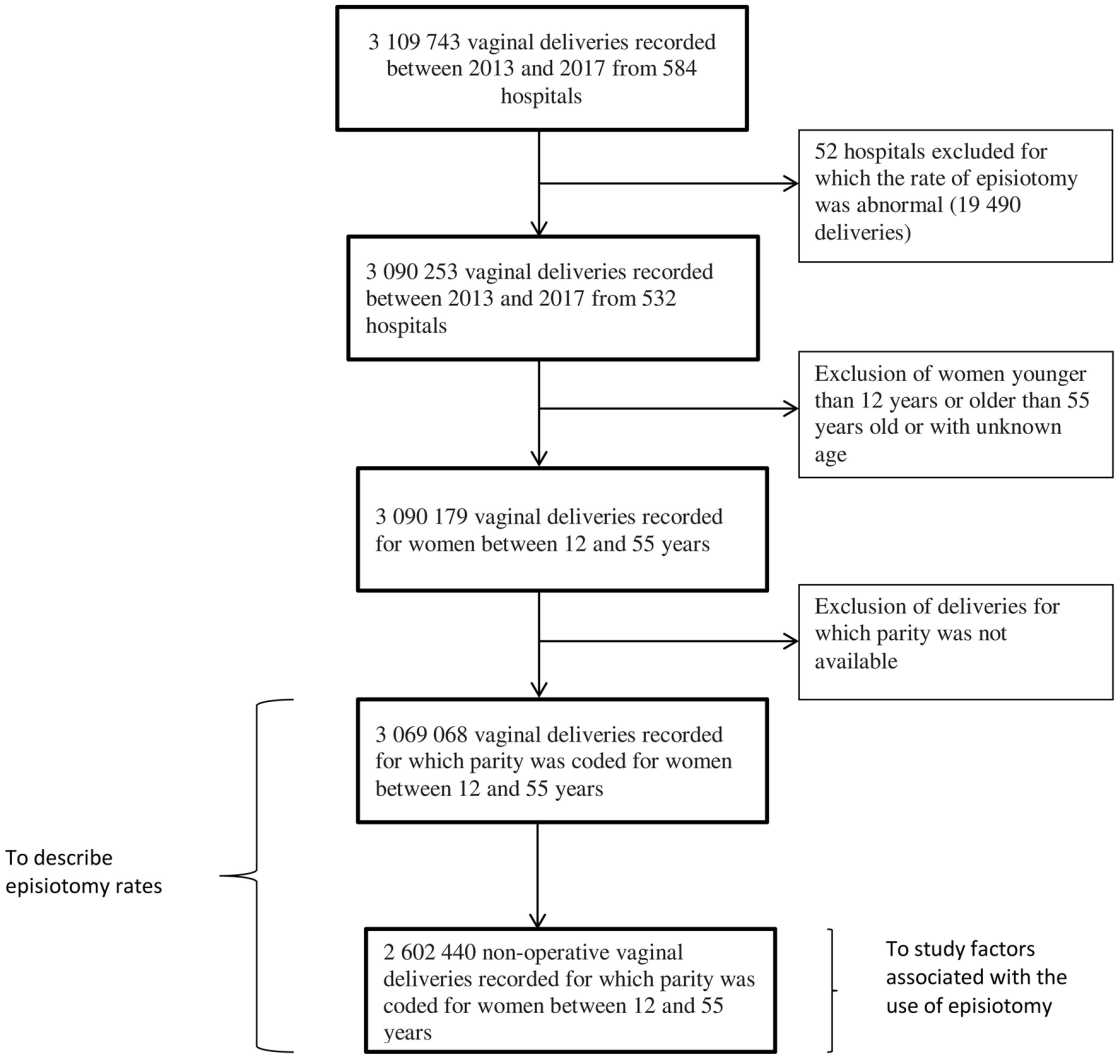
Shows the selection of the population.

Overall, 3,109 743 vaginal deliveries were recorded between 2013 and 2017 from 584 hospitals. We excluded 52 hospitals (19,490 deliveries; 0.63%) for which the rate of episiotomy was abnormal (i.e. where no episiotomy was coded or that have no maternity unit and had reported only one delivery), leading to 3,090,253 deliveries. Among these, 3,090,179 occurred in women between 12 and 55 years old. Finally, parity was available for 99.0% of these deliveries (N = 3,069,068). We then identified 2,602,440 non-operative vaginal deliveries. These figures are summarized in a flowchart (Fig. 1).

### Descriptive statistics

**Figure 2:**
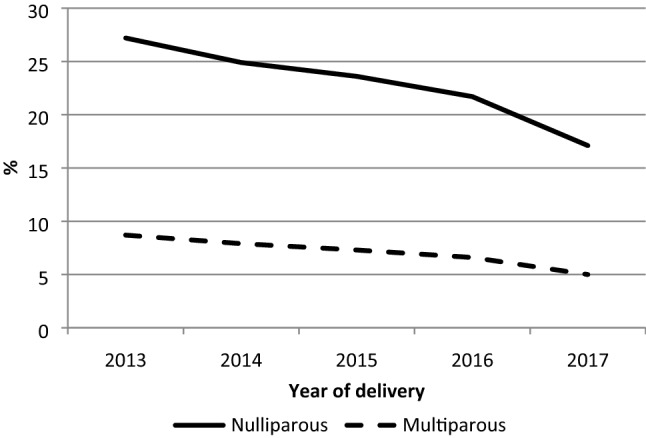
Shows the evolution of episiotomy rates in France from 2013 to 2017 by parity for vaginal deliveries without instruments.

Among the 3,069,068 vaginal deliveries included in our analyses, we observed an overall rate of episiotomy of 18.6% on the entire period (2013–2017) and a significant decrease (p < 0.01) of 7.3 percentage points in the use of episiotomy between 2013 (21.6%) and 2017 (14.3%) for all vaginal deliveries (rate of change of − 34%) (Table 1). More specifically, a significant reduction of 3.3 percentage points was observed between 2016 (17.7%) and 2017 (14.3%, p < 0.01), with a rate of change of − 19.2%. Concerning only non-operative vaginal deliveries (N = 2,602,440), the overall rate of episiotomy on the entire period (2013–2017) was 12.9% with a significant decrease (p < 0.01) between 2013 (15.5%) and 2017 (9.3%) and a rate of change of − 40% (Table 1). A reduction of 2.7 percentage points occurred between 2016 (12%) and 2017 (9.3%), with a rate of change of − 22.5%. Separating by parity (Supplementary Table S2 online), 27.2% of nulliparous and 8.7% of multiparous women had an episiotomy in 2013 (Fig. 2). The figures decreased steadily until 2017, with 17.1% for nulliparous (p < 0.01) and 5% for multiparous women (p < 0.01), showing a reduction of 10.1 percentages points (rate of change of − 37.1%) for nulliparous and of 3.7 percentages points (rate of change of − 42.5%) for multiparous deliveries.

**Table 1:** Episiotomy rates for all vaginal deliveries and for those without instruments in France 2013–2017.

Year of delivery	2013	2014	2015	2016	2017	p*	Overall
All vaginal deliveries	630,220	360,647	614,764	603,194	590,243		3,069,068
Episiotomy	135,894 (21.6%)	125,903 (20.0%)	116,539 (19.0%)	106,628 (17.7%)	84,521 (14.3%)	< 0.01	569,485 (18.6%)
Non operative-vaginal deliveries	535,182	535,234	521,267	510,648	500,109		2,602,440
Episiotomy	82,965 (15.5%)	75,375 (14.1%)	68,390 (13.1%)	61,202 (12.0%)	46,497 (9.3%)	< 0.01	334,429 (12.9%)

Data are presented as *n* (%).

*p-value for Cochran-Armitage test: < 0.01 for both nulliparous and multiparous for all vaginal deliveries and those without instrument.

### Comparative analysis (for non-operative vaginal deliveries)

Tables 2 and 3 present the women’s demographic and clinical characteristics as well as the frequency of episiotomy according to these characteristics, in the group of non-operative vaginal deliveries, for nulliparous and multiparous deliveries respectively.

**Table 2:** Comparative analyses for nulliparous and for non-operative vaginal deliveries.

	N (%)	Episiotomy	p-value*	Crude RR [95% CI]
Total	934,230	215,054 (23.02%)		
**Prematurity**			< 0.01	
No	867,471 (92.85%)	205,851 (23.73%)		Ref
Yes	66,759 (7.15%)	9,203 (13.79%)		0.58 [0.57–0.59]
**Hypertensive disorders**			< 0.01	
No	907,675 (97.16%)	209,614 (23.09%)		Ref
Yes	26,555 (2.84%)	5,440 (20.49%)		0.89 [0.87–0.91]
**Diabetes mellitus**			0.39	
No	875,603 (93.72%)	201,473 (23.01%)		Ref
Yes	58,627 (6.28%)	13,581 (23.17%)		1.01 [0.99–1.02]
**Age**			< 0.01	
< 20	52,223 (5.59%)	10,475 (20.06%)		0.86 [0.85–0.88]
20–29	571,855 (61.21%)	132,821 (23.23%)		Ref
30–39	297,028 (31.79%)	68,733 (23.14%)		1.00 [0.99–1.01]
≥ 40	13,124 (1.40%)	3,025 (23.05%)		0.99 [0.96–1.02]
**Multiple pregnancy**			0.02	
No	926,480 (99.17%)	213,185 (23.01%)		Ref
Yes	7,750 (0.83%)	1869 (24.12%)		1.05 [1.01–1.09]
**Presentation**^**a**^			< 0.01	
Cephalic	907,669 (97.91%)	208,212 (22.94%)		Ref
Breech	17,930 (1.93%)	4,750 (26.49%)		1.15 [1.13–1.18]
**Epidural analgesia**			< 0.01	
No	139,755 (14.96%)	25,415 (18.19%)		Ref
Yes	794,475 (85.04%)	189,639 (23.87%)		1.31 [1.30–1.33]
**Non-reassuring fetal heart**			< 0.01	
No	843,211 (90.26%)	187,566 (22.24%)		Ref
Yes	91,019 (9.74%)	27,488 (30.20%)		1.36 [1.34–1.37]
**Shoulder dystocia**			< 0.01	
No	926,750 (99.20%)	212,918 (22.97%)		Ref
Yes	7,480 (0.80%)	2,136 (28.56%)		1.24 [1.20–1.29]
**Meconium in amniotic fluid**			< 0.01	
No	848,289 (90.80%)	192,399 (22.68%)		Ref
Yes	85,941 (9.20%)	22,655 (26.36%)		1.16 [1.15–1.18]
**Teaching hospital**^**b**^			< 0.01	
No	699,870 (81.16%)	163,657 (23.38%)		Ref
Yes	162,433 (18.84%)	36,254 (22.32%)		0.95 [0.94–0.96]

Data are presented as n (%).

*Chi-2 test.

^a^Missing data < 1%.

^b^Missing data ≈ 7.5%.

**Table 3:** Comparative analyses for multiparous and for non-operative vaginal deliveries.

	N (%)	Episio	p-value*	Crude RR [95% CI]
Total	1,668,210	119,375 (7.16%)		
**Prematurity**			< 0.01	
No	1,586,707 (95.11%)	116,020 (7.31%)		Ref
Yes	81,503 (4.89%)	3,355 (4.12%)		0.56 [0.54–0.58]
**Hypertensive disorders**			< 0.01	
No	1,632,460 (97.86%)	117,352 (7.19%)		Ref
Yes	35,750 (2.14%)	2023 (5.66%)		0.79 [0.75–0.82]
**Diabetes mellitus**			< 0.01	
No	1,514,139 (90.76%)	108,719 (7.18%)		Ref
Yes	154,071 (9.24%)	10,656 (6.92%)		0.96 [0.94–0.98]
**Age**			< 0.01	
< 20	11,310 (0.68%)	576 (5.09%)		0.76 [0.70–0.83]
20–29	604,236 (36.22%)	40,306 (6.67%)		Ref
30–39	973,337 (58.35%)	73,050 (7.51%)		1.13 [1.11–1.14]
≥ 40	79,327 (4.76%)	5,443 (6.86%)		1.03 [1.01–1.06]
**Multiple pregnancy**			< 0.01	
No	1,652,764 (99.07%)	117,956 (7.14%)		Ref
Yes	15,446 (0.93%)	1,419 (9.19%)		1.29 [1.22–1.35]
**Presentation**^**a**^			< 0.01	
Cephalic	1,640,454 (99.12%)	116,930 (7.13%)		Ref
Breech	10,886 (0.66%)	830 (7.62%)		1.07 [1.01–1.14]
**Epidural analgesia**			< 0.01	
No	474,134 (28.42%)	23,000 (4.85%)		Ref
Yes	1,194,076 (71.58%)	96,375 (8.07%)		1.66 [1.64–1.69]
**Non-reassuring fetal heart**			< 0.01	
No	1,554,645 (93.19%)	107,182 (6.89%)		Ref
Yes	113,565 (6.81%)	12,193 (10.74%)		1.56 [1.53–1.59]
**Shoulder dystocia**			< 0.01	
No	1,644,612 (98.59%)	116,944 (7.11%)		Ref
Yes	23,598 (1.41%)	2,431 (10.30%)		1.45 [1.39–1.50]
**Meconium in amniotic fluid**			< 0.01	
No	1,533,212 (91.91%)	108,225 (7.06%)		Ref
Yes	134,998 (8.09%)	11,150 (8.26%)		1.17 [1.15–1.19]
**Scarred uterus**			< 0.01	
No	1,571,053 (94.18%)	103,848 (6.61%)		Ref
Yes	97,157 (5.82%)	15,527 (15.98%)		2.42 [2.38–2.46]
**Teaching hospital**^**b**^			< 0.01	
No	1,265,094 (81.87%)	93,058 (7.36%)		Ref
Yes	280,223 (18.13%)	17,897 (6.39%)		0.87 [0.85–0.88]

Data are presented as n (%).

*Chi-2 test.

^a^Missing data = 1%.

^b^Missing data ≈ 7.5%.

Overall, we found a rate of episiotomy of 23.1% for nulliparous (Table 2) and of 7.2% for multiparous deliveries (Table 3). Whatever the parity, we found an association between almost each characteristics and episiotomy. In particular, we found that there were more episiotomies for deliveries with epidural analgesia, NRFHR, shoulder dystocia, and meconium in amniotic fluid than for deliveries without these clinical characteristics. We also found that the rate of episiotomy was higher in cases of breech presentations or multiple pregnancies. On the contrary, this rate was lower for prematurity and for deliveries performed in teaching hospitals. For multiparous deliveries, the rate of episiotomy varied according to age and was 2.5 times higher for women with a scarred uterus than those without.

We also compared the rates of each characteristic between the episiotomy group and the non-episiotomy group. Results are presented in the Supplementary material (Supplementary Tables S3 and S4 online).

### Multilevel analyses (for non-operative vaginal deliveries)

For nulliparous women (Table 4), after adjustment on hypertensive disorders, gestational diabetes, hospital status and year of delivery, whatever the age group, the risk factors for episiotomy were epidural analgesia (1.22 ≤ RR ≤ 1.35) and NRFHR (1.33 ≤ RR ≤ 1.41). When we also adjusted on newborn weight for singleton deliveries, we found that the same factors, in addition to the newborn weight higher than 4,000 g, significantly increased the risk of episiotomy (1.14 ≤ RR ≤ 1.73). Prematurity decreased the risk of episiotomy (RR between 0.47 [0.45–0.50] for women between 30 and 39 years and 0.58 [0.52–0.65] for those younger than 20 years). The type of pregnancy (multiple/single) was not associated with episiotomy.

**Table 4:** Factors associated with episiotomy for nulliparous and non-operative vaginal deliveries according to maternal age (hierarchical regression adjusted on the year of delivery, hospital status, hypertensive disorders and gestational diabetes).

	< 20 years			20–29 years			30–39 years			≥ 40 years		
	RR	CI−	CI+	RR	CI−	CI+	RR	CI−	CI+	RR	CI−	CI+
**All deliveries**												
Prematurity	0.62	0.57	0.69	0.59	0.56	0.62	0.55	0.51	0.59	0.53	0.45	0.62
Epidural analgesia	1.35	1.14	1.61	1.23	1.14	1.33	1.31	1.22	1.42	1.22	1.06	1.39
Non-reassuring fetal heart	1.41	1.30	1.53	1.36	1.29	1.43	1.33	1.26	1.40	1.34	1.20	1.49
Meconium in amniotic fluid	1.10	1.01	1.19	1.15	1.11	1.2	1.16	1.11	1.21	1.36	1.23	1.50
Shoulder dystocia	–	–	–	1.21	1.13	1.3	1.13	1.03	1.23	–	–	–
Multiple pregnancy	–	–	–	–	–	–	–	–	–	–	–	–
Breech presentation	–	–	–	1.35	1.24	1.48	1.35	1.17	1.54	–	–	–
**Singleton deliveries**												
Prematurity	0.58	0.52	0.65	0.53	0.51	0.55	0.47	0.45	0.50	0.49	0.40	0.61
Epidural analgesia	1.33	1.23	1.44	1.31	1.28	1.34	1.44	1.40	1.49	1.31	1.14	1.50
Non-reassuring fetal heart	1.77	1.63	1.92	1.56	1.52	1.59	1.48	1.43	1.52	1.55	1.34	1.78
Meconium in amniotic fluid	1.10	1.01	1.20	1.24	1.21	1.27	1.23	1.19	1.27	1.59	1.39	1.83
Shoulder dystocia	–	–	–	1.24	1.15	1.34	–	–	–	–	–	–
Newborn weight (ref < 4,000 g)	1.73	1.51	1.98	1.62	1.56	1.67	1.14	1.02	1.28	1.40	1.09	1.82
Breech presentation	–	–	–	1.65	1.56	1.74	1.58	1.48	1.68	–	–	–

RR: Relative risk; CI: confidence interval.

For multiparous women (Table 5), after adjustment on hypertensive disorders, gestational diabetes, hospital status and year of delivery, whatever the age group, scared uterus (1.97 ≤ OR ≤ 4.13), NRFHR (1.57 ≤ OR ≤ 1.86), shoulder dystocia (1.48 ≤ OR ≤ 1.95) and epidural analgesia (1.58 ≤ OR ≤ 1.77) were most strongly associated with episiotomy. For the groups of women aged 20 years or older, multiple pregnancy (1.71 ≤ OR ≤ 4.05) was associated with episiotomy. When we adjusted on newborn weight for singleton deliveries, we found the same associated factors, and newborn weight higher than 4,000 g significantly increased the risk of episiotomy (1.27 ≤ OR ≤ 1.63). Furthermore, whatever the age group, prematurity reduced the risk of episiotomy by half (e.g. OR = 0.41 [0.34–0.49] for women more than 40 years).

**Table 5:** Factors associated with episiotomy for multipara and non-operative vaginal deliveries according to maternal age (hierarchical regression adjusted on the year of delivery, hospital status, hypertensive disorders and gestational diabetes).

	< 20 years			20–29 years			30–39 years			≥ 40 years		
	OR	CI−	CI+	OR	CI−	CI+	OR	CI−	CI+	OR	CI−	CI+
**All deliveries**												
Prematurity	0.54	0.36	0.80	0.52	0.49	0.56	0.54	0.51	0.56	0.41	0.34	0.49
Epidural analgesia	1.77	1.41	2.22	1.58	1.54	1.63	1.60	1.57	1.64	1.73	1.60	1.87
Non-reassuring fetal heart	1.86	1.36	2.53	1.60	1.54	1.67	1.57	1.53	1.62	1.57	1.43	1.73
Meconium in amniotic fluid	1.64	1.19	2.27	1.25	1.21	1.30	1.19	1.15	1.23	1.21	1.10	1.33
Shoulder dystocia	1.95	1.06	3.60	1.55	1.43	1.68	1.48	1.39	1.57	1.75	1.44	2.14
Scarred uterus	4.13	3.08	5.56	3.33	3.22	3.45	2.60	2.53	2.67	1.97	1.79	2.16
Multiple pregnancy	–	–	–	1.98	1.53	2.56	1.71	1.45	2.03	4.05	2.44	6.70
Breech presentation	–	–	–	–	–	–	–	–	–	–	–	–
**Singleton deliveries**												
Prematurity	0.48	0.30	0.77	0.54	0.50	0.58	0.56	0.52	0.59	0.42	0.34	0.52
Epidural analgesia	1.68	1.32	2.15	1.57	1.52	1.62	1.58	1.55	1.62	1.73	1.60	1.87
Non-reassuring fetal heart	1.53	1.06	2.20	1.60	1.53	1.66	1.60	1.55	1.64	1.59	1.44	1.76
Meconium in amniotic fluid	1.50	1.04	2.15	1.24	1.19	1.29	1.17	1.14	1.21	1.18	1.07	1.31
Shoulder dystocia	–	–	–	1.26	1.16	1.38	1.27	1.19	1.36	1.54	1.24	1.92
Scarred uterus	4.81	3.47	6.62	3.40	3.28	3.52	2.64	2.57	2.71	2.02	1.83	2.23
Newborn weight (ref < 4,000 g)	1.63	1.08	2.46	1.63	1.57	1.69	1.49	1.45	1.53	1.27	1.14	1.40
Breech presentation	–	–	–	–	–	–	–	–	–	–	–	–

OR: Odds ratio; CI: confidence interval.

## Discussion

Episiotomy was used in 14.3% of French vaginal deliveries in 2017, thus indicating a significant and continuing decline from 2013 (− 7.3 percentage points and a rate of change of − 34%). Analysis focusing on “non-operative vaginal delivery” revealed a similar decline of − 6.2 percentage points (from 15.5% in 2013 to 9.3% in 2017) with a rate of change of − 40%. These observations are consistent with the French effort to reduce the practice of episiotomy that was initiated in 1996^9^. Moreover, since 2005 (overall episiotomy rate of 41.3%^9^, which was the year that the French guidelines on episiotomy were published^8^, the rate of episiotomy significantly decreased at national level with a rate of 19.9% in 2014^28^. Our study, covering the period up to 2017, shows that this decrease is still relevant.

First, the French episiotomy rate in 2017 for all vaginal deliveries was 14.3%. This rate is far lower than the clinical practice recommendations issued by the French national college of obstetricians and gynecologists (CNGOF) in 2005 (episiotomy rate < 30%)^8,9^, and it confirms that France holds an intermediate position in Europe and in the world (among highly industrialized, medicalized countries which have promoted the institutionalization of childbirth)^4^. The current episiotomy rate in France is close to the rate reported in the UK in 2011/2012 (15.20%)^3^. Though far lower than rates in Portugal (72.9%), Spain (43%) in 2010 or Slovenia (31.3%) in 2013, the French rate is still far higher than in Denmark (4.9%), Sweden (6.6%) or the United States (9.4%) in 2011^3,38^. In our study, the episiotomy rates of 14.3% in 2017 and 17.6% in 2016 for all vaginal deliveries are lower than the most recently published estimation (20.1% in 2016)^39^. This difference may be explained by the research methodology. The national perinatal survey included representative samples from nearly all maternity hospitals and over 1 week, while our study was a population-based study from data coding based on data from the entire year.

Between 2013 and 2017, the observed reduction in episiotomies for all vaginal deliveries was − 34%, with a more pronounced reduction (− 40%) for non-operative vaginal deliveries. The reasons for this decline are multifactorial. Some strategies that were implemented may have facilitated the reduction of episiotomy rates. For instance, the diffusion of institutional annual^27^ or monthly episiotomy rates^40^, the diffusion of individual annual episiotomy rates (specific to each operator), clinical audits based on patient records, retrospective analysis of the decisions process^27,41^, the obligation to mention the indication that led to the episiotomy on the operative report^40^, additional training^27,41^ and regular reminders of clinical practice recommendations^27,40,41^ have probably all contributed to the decreasing episiotomy rate. Over time, encouraged by the CNGOF as well as political and associative groups, each institution has relied on some of these initiatives to reduce episiotomy rates. The effectiveness of this methodology has also been evaluated positively in the international literature^15,42,43^. Finally, the reduction that we observed between 2016 and 2017 boosted the general downward trend (rate of change of about − 20%), showing the potential impact of the national debate about episiotomy practice in France^29,44^. The reduction in the episiotomy rate deserves to be compared to the obstetric anal sphincter injuries (OASIS) rate considering that there is concern about an increase in OASIS associated with a decrease in episiotomies^4,9,28,45–48^. Based on our previous study^28^, we found that severe perineal tears (third and fourth degrees) significantly increased in women who had non-operative vaginal deliveries. These results were consistent with those of the Euro-peristat project, which described an increase in the rate of severe perineal tears for all vaginal deliveries between 2004 and 2010 in all European countries, except Germany and Norway^46^.

Second, among all of the factors available in the PMSI database for non-operative vaginal deliveries, we focused on the variables associated with episiotomy that were major risk factors according to the scientific literature. This includes multiple pregnancies, breech vaginal deliveries, epidural analgesia, NRFHR, and newborn weight > 4,000 g^7,20,23–26,49^. In this study, the results obtained in both regression models once again underline that NRFHR, epidural analgesia, and newborn weight > 4,000 g are factors associated with episiotomy for nulliparous deliveries. For multiparous deliveries, the factors associated with episiotomy use were NRFHR, shoulder dystocia, epidural analgesia, multiple pregnancy, and newborn weight > 4,000 g. All of these results are in accordance with the existing literature^16^. We noticed that NRFHR is one of the most important risk factors for episiotomies, which indicates that the baby’s safety is prevalent for French midwifes and obstetricians. The strong association between epidural analgesia and episiotomy may be the reflection of the widespread use of epidurals in France (83.8% of women in 2016)^11,50^. However, previous publications from countries with lower rates of epidural analgesia showed an association between epidural analgesia and episiotomy^51^ or unclear association^52^. In the case of epidural analgesia, the pushing efforts are less effective due to the anesthetic and the resulting lack of sensitivity. At the beginning of labor, women may start pushing too early or in an inappropriate way, while at the end of labor, the urge to push will be inhibited. These inappropriate pushing efforts can then lead to instrumental extraction or an episiotomy. We also wish to underline that multiple pregnancy was a risk factor for multiparous women only. This observation was also retrieved from a study from the AUDIPOG Network^22^ (Association des Utilisateurs de Dossiers Informatisés en Pédiatrie, Obstétrique et Gynécologie), which is a group of public and private maternity clinics from all over France. This result may be explained by the fact that multiple pregnancies can lead to maneuvers that increase the risk of episiotomy.

One of our analyses was restricted to singleton delivery in order to adjust on newborn weight. Though the linkage between the mother and the newborn has been available in the French PMSI database since 2013, it is difficult to link newborns from multiple pregnancies as they can have the same identification and be hardly differentiable, especially when they have the same gender. Newborn weight higher than 4,000 g was found to be a main risk factor. This result is consistent with the scientific literature^16^.

One of the strengths of this population-based study lies in the completeness of the data from French hospitals and the fact that, in France, 99.6% of births take place in hospitals^53,54^. Thus, this study covers almost all women who gave birth in France between 2013 and 2017. Concerning the risk factors, we focused on non-operative vaginal deliveries, which is a subgroup of deliveries considered to be at a "reduced-risk" of complications during delivery and of episiotomy worldwide, which means that the results of this study can potentially be extrapolated to other countries. Another strength is the methodology used in our study, which included two types of hierarchical regressions according to the rarity of episiotomy^37^. Finally, the quality of the database used in this study has been evaluated and confirmed in recent studies^34,53,55–57^. In particular, the reliability of perinatal indicators was assessed and confirmed in a 2014 validation study that aimed to evaluate the metrological quality of hospital discharge abstracts for perinatal indicators^34^. For vaginal deliveries, the positive predictive value (PPV) was 99.5% [99.3–99.7] and the sensitivity (Se) was 99.6% [99.4–99.8]. For parity, in women with vaginal deliveries, the PPV was 93.3% [92.3–94.3] for nulliparous women and 95.5% [94.7–96.3] for multiparous women, and the Se was 92.4% [91.3–93.5] and 95.5% [94.7–96.3], respectively. For episiotomy performed during vaginal deliveries (with or without instruments), the PPV was 90.1% [88.9–91.3] and the Se was 86.0% [84.6–87.4].

We acknowledge that our study has certain limits. Even if the reliability of episiotomy appears to be good for all vaginal deliveries, episiotomies associated with operative vaginal deliveries may not be systematically coded by the operator. This under-estimation is not very high (less than 10%) and we were able to estimate rates for all vaginal deliveries (taking into account these 10% of missing data, a corrected estimation of the overall rate of episiotomy for all vaginal deliveries would be 19.3% instead of 18.6%). However our study is exposed to data loss bias when conducting analyses on risk factors. For this reason, we studied only risk factors for non-operative vaginal deliveries (for which our previous study showed no substantial lack of coding). Moreover, we know that we could not include all factors associated with episiotomy such as length of labor, length of expulsive stage, induction of labor or previous episiotomy practice for multipara, but we used all the individual and clinical factors available in our database, which contains most of the factors found in the literature. A potential under-detection-related bias is another limitation for comorbidities such as diabetes and hypertensive disorders^33^. Coding practices may vary among institutions since the people who perform the coding of diagnoses can be clinicians or information system technicians. Nevertheless, coding quality is checked in a standardized manner by medical information professionals in each hospital to correct diagnoses and improve the level of comorbidity recording (internal quality assessment).

## Conclusion

In conclusion, the use of episiotomy for non-operative vaginal deliveries has fallen steadily in France, from 15.5% in 2013 to 9.3% in 2017, and episiotomy is now used in less than 15% of deliveries nationwide. Despite the reduction in episiotomy over the last few years, our study highlights that the factors associated with episiotomy use for non-operative vaginal deliveries, such as multiple pregnancies, epidural analgesia, and NRFHR, were broadly the same throughout the study period and similar to the literature. These factors have not decreased with time, which suggests that the decrease in episiotomies in France is not the result of specific factors but probably related to several care improvement strategies that have been relayed by hospital teams and perinatal networks: continuous training, clinical auditing with feedback, and communication about internal practices. Nonetheless, this reduction should not obscure the importance of maintaining appropriate communication between the caregiver and the pregnant woman (information-explanation-consent), whether the episiotomy is needed in an emergency context or not.

## Supplementary information

Supplementary Information 1.

Supplementary Information 2.

## Data Availability

The use of these data by our department was approved by the National Committee for data protection. We are not allowed to transmit these data. PMSI data are available for researchers who meet the criteria for access to these French confidential data (this access is submitted to the approval of the National Committee for data protection) from the national agency for the management of hospitalization (ATIH—Agence technique de l'information sur l'hospitalisation). Address: Agence technique de l'information sur l'hospitalisation, 117 boulevard Marius Vivier Merle, 69329 Lyon Cedex 03. The website for researchers to contact the ATIH is: https://www.atih.sante.fr/nous-contacter.
