# Author Correction: Episiotomy practices in France: epidemiology and risk factors in non-operative vaginal deliveries

**DOI:** 10.1038/s41598-021-85717-1

**Published:** 2021-03-19

**Authors:** Christophe Clesse, Jonathan Cottenet, Joelle Lighezzolo-Alnot, Karine Goueslard, Michele Scheffler, Paul Sagot, Catherine Quantin

**Affiliations:** 1 Centre for Psychiatry, Wolfson Institute of Preventive Medicine, Barts & The London School of Medicine & Dentistry, Queen Mary, University of London, Old Anatomy Building Charterhouse Square, London, EC1M 6BQ UK; 2 Interpsy Laboratory (EA 4432), Universite de Lorraine - Campus Lettres Et Sciences Humaines, Nancy, France; 3 Majorelle Polyclinic, Nancy, France; 4 Biostatistics and Bioinformatics (DIM), University Hospital, University of Burgundy and Franche-Comté, Dijon, France; 5 Psychologie Clinique, Interpsy Laboratory (EA4432), Lorraine University, Nancy, France; 6 Obstetricial Gynecologist, Endocrinologist, Gynecologist, The FNCGM (National Federation of Gynecology Medical Colleges), Cabinet de Gynécologie Médicale Et Obstétrique, 21 avenue Foch, 54000 Nancy, France; 7 Department of Obstetrics and Gynecology, University Hospital, Dijon, France; 8 Inserm, CIC 1432, Clinical Investigation Center, Clinical Epidemiology/Clinical Trials Unit, Dijon University Hospital, Dijon, France; 9 Biostatistics, Biomathematics, Pharmacoepidemiology and Infectious Diseases (B2PHI), INSERM, UVSQ, Institut Pasteur, Université Paris-Saclay, Paris, France

Correction to: *Scientific Reports* 10.1038/s41598-020-70881-7, published online 19 November 2020

The original version of this Article contained errors in the spelling of the authors Christophe Clesse, Jonathan Cottenet, Joelle Lighezzolo-Alnot, Karine Goueslard, Michele Scheffler, Paul Sagot and Catherine Quantin, which were incorrectly given as Clesse Christophe, Cottenet Jonathan, Lighezzolo-Alnot Joelle, Goueslard Karine, Scheffler Michele, Sagot Paul and Quantin Catherine.

These errors have now been corrected in the PDF and HTML versions of the Article.

