# Supplementary material for: Episiotomy practices in France: epidemiology and risk factors in non-operative vaginal deliveries
Source: Sci Rep. 2020 Nov 19;10:20208. doi: 10.1038/s41598-020-70881-7 (PMC7677317; doi:10.1038/s41598-020-70881-7)
Supplement: Supplementary file 1 — Supplementary Information 1. [file 41598_2020_70881_MOESM1_ESM.docx]

Episiotomy practices in France: epidemiology and risk factors in non-operative vaginal deliveries

Clesse C, Cottenet J, Lighezzolo-Alnot J, Goueslard K, Scheffler M, Sagot P, Quantin C

**Supplementary Table S1: ICD-10 and CCMP codes of recorded items**

| Studied variables | ICD10 codes | CCMP codes |
| --- | --- | --- |
| Vaginal delivery without instrument | O80 - O840 | JQGD001 - JQGD002 - JQGD003 - JQGD004 - JQGD005 - JQGD007 - JQGD008 - JQGD010 - JQGD012 - JQGD013 |
| Vaginal delivery with instrument | O81 - O841 | JQGD006 - JQGD009 - JQGD011 |
| Nulliparous |  | JQGD002 - JQGD003 - JQGD004 - JQGD010 - JQGD013 |
| Multiparous |  | JQGD001 - JQGD005 - JQGD007 - JQGD008 - JQGD012 |
| Single pregnancy | Z370 - Z371 |  |
| Multiple pregnancy | Z372 - Z373 - Z374 - Z375 - Z376 - Z377 |  |
| Cephalic presentation for vaginal delivery |  | JQGD006 - JQGD009 - JQGD010 - JQGD012 |
| Breech presentation for vaginal delivery | O321 - O641 - O801 - O830 - O831 | JQGD001 - JQGD003 - JQGD004 - JQGD005 - JQGD008 - JQGD011 - JQGD013 |
| Transverse presentation for vaginal delivery | O642 - O643 - O644 - O645 - O648 - O649 |  |
| Diabetes Mellitus | O24 - E10 - E11 - E12 - E13 - E14 |  |
| Hypertensive disorders | O10 - O11 - O13 - O14 - O15 - I10 - I11 - I12 - I13 - I15 |  |
| Epidural analgesia |  | AFLB010 |
| Non-reassuring fetal heart rate | O680 |  |
| Shoulder dystocia | O644 - O660 |  |
| Meconium in amniotic fluid | O681 - O682 |  |
| Scarred uterus | O342 - O757 |  |

**Supplementary Table S2: Episiotomy rates by parity for all vaginal deliveries and for those without instruments in France 2013-2017**

| Year of delivery | 2013 | | 2014 | | 2015 | | 2016 | | 2017 | | p* |
| --- | --- | --- | --- | --- | --- | --- | --- | --- | --- | --- | --- |
| All vaginal deliveries | Nu | M | Nu | M | Nu | M | Nu | M | Nu | M |  |
|  | N=269 145 | N=361 075 | N=266 897 | N=363 750 | N=258 145 | N=356 619 | 252 149 | N=351 045 | 246 098 | N=344 145 |  |
| Episiotomy | 97 027 | 38 867 | 89 763 | 36 140 | 83 609 | 32 930 | 76 795 | 29 833 | 61 781 | 22 740 |  |
|  | (36.1%) | (10.8%) | (33.6%) | (9.9%) | (32.4%) | (9.2%) | (30.5%) | (8.5%) | (25.1%) | (6.6%) | <0.01 |
| Vaginal deliveries without instrument | Nu | M | Nu | M | Nu | M | Nu | M | Nu | M |  |
|  | N=195 674 | N=339 508 | N=193 649 | N=341 585 | N=186 308 | N=334 959 | N=181 509 | N=329 139 | N=177 090 | N=323 019 |  |
| Episiotomy | 53 276 | 29 689 | 48 252 | 27 123 | 43 871 | 24 519 | 39 374 | 21 828 | 30 281 | 16 216 |  |
|  | (27.2%) | (8.7%) | (24.9%) | (7.9%) | (23.6%) | (7.3%) | (21.7%) | (6.6%) | (17.1%) | (5.0%) | <0.01 |

Data are presented as *n* (%)

Nu = Nullipara

M = Multipara

*p-value for Cochran-Armitage test: <0.01 for both nulliparous and multiparous for all vaginal deliveries and those without instrument

**Supplementary Table S3: Comparative analyses for nulliparous and for non-operative vaginal deliveries**

|  | Episiotomy (n=215 054) | Without episiotomy (n=719 176) | *p-value** |
| --- | --- | --- | --- |
| Prematurity  Hypertensive disorders  Diabetes Mellitus | 9203 (4.28%)  5440 (2.53%)  13 581 (6.32%) | 57 556 (8.00%)  21 115 (2.94%)  45 046 (6.26%) | <0.01  <0.01  0.39 |
| Age  <20  20-29  30-39  >40 | 10 475 (4.87%)  132 821 (61.76%)  68 733 (31.96%)  3025 (1.41%) | 41 748 (5.80%)  439 034 (61.05%)  228 295 (31.74%)  10 099 (1.40%) | <0.01 |
| Multiple pregnancy | 1869 (0.97%) | 5881 (0.82%) | 0.02 |
| Presentation ^a^  Cephalic  Breech | 208 212 (97.56%)  4750 (2.22%) | 699 457 (98.01%)  13 180 (1.85%) | <0.01 |
| Epidural analgesia | 189 639 (88.18%) | 604 836 (84.10%) | <0.01 |
| Non-reassuring fetal heart | 27 488 (12.78%) | 63 531 (8.83%) | <0.01 |
| Shoulder dystocia | 2136 (0.99%) | 5344 (0.74%) | <0.01 |
| Meconium in amniotic fluid | 22 655 (10.53%) | 63 286 (8.80%) | <0.01 |
| Teaching hospital ^b^ | 36 254 (18.14%) | 126 179 (19.05%) | <0.01 |

Data are presented as n (%)

* Chi-2 test

a: Missing data < 1%

b: Missing data ≈ 7.5%

**Supplementary Table S4: Comparative analyses for multiparous and for non-operative vaginal deliveries**

|  | Episiotomy (n=119 375) | Without episiotomy (n=1 548 835) | *p-value** |
| --- | --- | --- | --- |
| Prematurity  Hypertensive disorders  Diabetes Mellitus | 3355 (2.81%)  2023 (1.69%)  10 656 (8.93%) | 78 148 (5.05%)  33 727 (2.18%)  143 415 (9.26%) | <0.01  <0.01  <0.01 |
| Age  <20  20-29  30-39  >40 | 576 (0.48%)  40 306 (33.76%)  73 050 (61.19%)  5443 (4.56%) | 10 734 (0.69%)  563 930 (36.41%)  900 287 (58.13%)  73 884 (4.77%) | <0.01 |
| Multiple pregnancy | 1419 (1.19%) | 14 027 (0.91%) | <0.01 |
| Presentation ^a^  Cephalic  Breech | 116 930 (98.95%)  830 (0.70%) | 1 523 524 (99.14%)  10 056 (0.65%) | <0.01 |
| Epidural analgesia | 96 375 (80.73%) | 1 097 701 (70.87%) | <0.01 |
| Non-reassuring fetal heart | 12 193 (10.21%) | 101 372 (6.55%) | <0.01 |
| Shoulder dystocia | 2431 (2.04%) | 21 167 (1.37%) | <0.01 |
| Meconium in amniotic fluid | 11 150 (9.34%) | 123 848 (8.00%) | <0.01 |
| Scarred uterus | 15 527 (13.01%) | 81 630 (5.27%) | <0.01 |
| Teaching hospital ^b^ | 17 897 (16.13%) | 262 326 (18.29%) | <0.01 |

Data are presented as n (%)

* Chi-2 test

a: Missing data = 1%

b: Missing data ≈ 7.5%
